# The Effects of the S1P Receptor Agonist Fingolimod (FTY720) on Central and Peripheral Myelin in Twitcher Mice

**DOI:** 10.3390/biomedicines12030594

**Published:** 2024-03-06

**Authors:** Sibylle Béchet, Kumlesh K. Dev

**Affiliations:** Drug Development, School of Medicine, Trinity College Dublin, Dublin, Ireland

**Keywords:** globoid cell leukodystrophy, Krabbe’s disease, myelination, FTY720, S1P receptors

## Abstract

Krabbe’s disease (KD) is caused by mutations in the lysosomal enzyme galactocerebrosidase and is associated with psychosine toxicity. The sphingosine 1-phosphate receptor (S1PR) agonist fingolimod (FTY720) attenuates psychosine-induced cell death of human astrocytes, demyelination in cerebellar slices, as well as demyelination in the central nervous system of twitcher mice. Psychosine also accumulates in the peripheral nervous system in twitcher mice; however, effects of fingolimod on this peripheral myelin have not been examined. The aim of this study was to investigate the effects of fingolimod administration on peripheral and central markers of myelination. Here, we report that fingolimod administration (1 mg/kg/day) from postnatal day 5 (PND) onwards did not alter peripheral demyelination in the sciatic nerve of twitcher mice, despite significantly reducing myelin debris, glial reactivity, and neuronal damage in the cerebellum. We also find fingolimod administration improves twitching and mobility scores in twitcher mice. Importantly, we find that fingolimod significantly increases the lifespan of twitcher mice by approximately 5 days. These findings suggest differential effects of fingolimod on peripheral and central neuropathy in twitcher mice, which may explain its modest efficacy on behavior and lifespan.

## 1. Introduction

The family of sphingosine 1-phosphate receptors (S1PR) are important mediators of many cellular functions, including cells in the immune and central nervous systems [1,2]. S1PRs are targets of the first oral drug used in a relapsing remitting form of multiple sclerosis, namely, fingolimod (FTY720/Gilenya®) [2]. In addition to their use in multiple sclerosis, S1PR-targeting (or S1PR-related) drugs such as fingolimod and siponimod have been examined for efficacy in several neurodegenerative diseases, in both, pre-clinical and clinical studies [2]. Given the suggested value of targeting S1PRs in a variety of neurodegenerative diseases, we examined the use of fingolimod in experimental models of Krabbe’s disease (KD). Krabbe’s disease is a neurodegenerative globoid cell leukodystrophy that is invariably fatal within the first two years of life [3]. This genetic disease is associated with mutations in the gene encoding lysosomal enzyme galactocerebrosidase, leading to the accumulation of lipid metabolites, including psychosine.

Previously, we have shown that psychosine causes human and mouse astrocyte toxicity in dissociated cell cultures, and demyelination in mouse organotypic slice cultures [4]. We have shown also that fingolimod attenuates psychosine-, as well as H_2_O_2_-, lysolecithin- and splenocyte-induced demyelination [1,2,4,5,6,7]. In addition, we found that fingolimod attenuates demyelination and inflammation in twitcher mice, which is a murine model of KD [8]. Twitcher mice have a spontaneous occurring mutation in the gene encoded galactocerebrosidase, thus making them a genetically equivalent model to human KD [3]. Beneficial effects of fingolimod on remyelination and inflammatory levels included a significant increase in the level of myelin basic protein (MBP) accompanied by decreases in astrogliosis and microglia reactivity in twitcher mice [8]. The therapeutic administration of fingolimod at postnatal day 20 only modestly increased life span of twitcher mice, with neurologic symptoms such as ataxia and paralysis ultimately prevailing. 

As well as accumulating in myelinating oligodendrocytes, pyschosine can also accumulate in Schwann cells in the peripheral nervous system (PNS), causing cytotoxicity, cell death, and demyelination in twitcher mice [9,10,11,12]. The effects of fingolimod on peripheral neuropathy have previously never been examined in twitcher mice. Therefore, the aim of the present study was to examine the effects of fingolimod administration in twitcher mice from postnatal day 5 onwards on central and peripheral markers of myelination and inflammation. Here, we present the effects of fingolimod on demyelination in the sciatic nerve in twitcher animals.

## 2. Materials and Methods

### 2.1. Animal Studies

All experiments were conducted in accordance with European Union (EU) guidelines and authorized by the Health Products Regulatory Authority (HPRA). A breeding colony of heterozygous twitcher mice was established in a pathogen free environment in the Comparative Medicine Unit, Trinity College Dublin, using mice obtained from the Jackson Laboratory and maintained on a C57BL/6j genetic background (C57BL/6J-twi with C57BL/6J-twi), as previously indicated [8]. Homozygous, heterozygous and wildtype animals were identified using genotyping. Heterozygous animals were used for breeding purposes. Fingolimod (FTY720) (SML0700, Sigma-Aldrich, Darmstadt, Germany) was administered to both male and female wildtype and homozygous twitcher mice via suckling pipette at a concentration of 1 mg/kg/day. Due to overt phenotypic emergence in twitcher mice, the genotype of animals used in this study became evident during the study. Thus, no genotype-based blinding was possible, and no blinding was included at the level of drug treatment. Given the severity of this animal model and in line with ethical considerations, we did not separate male and female animals to avoid increasing animal numbers. In this study, both male and female twitcher animals were used, where we observed no overt differences between these groups. All data were randomized, and analysis was performed with data blinded to both genotype and drug treatment as before [8,13].

### 2.2. Animal Behaviour

Behavioral observations were taken daily up to 3 times per day and body weight was measured daily. An observational scale was used to classify both the frequency and the severity of twitching during disease progression. The twitching and immobility scoring was conducted as previously published [8]. Locomotion was also assessed using the Open Field Maze (OFM) to investigate more subtle changes in behavior at an early age and before severe immobility and twitching symptoms emerge. Animals were naive to experiments. Animals were placed in the OFM for 5 min on postnatal day (PND) 22 for habituation. All animals were behaviourally assessed in an OFM on PND25, PND28 and PND30. All experiments were carried out following the same protocol, at the same time of day, with the same conditions of luminosity. The time of day for behavioral analysis was between 8 a.m. and 12 p.m. For consistency, each animal was tested at the same time of day for habituation and each assessment. A video camera connected to a computer was placed above the OFM. After each session, the OFM was cleaned with 70% Ethanol. Each testing session was recorded for later analysis with ANY-Maze tracking software 60000 (Stoelting). The following parameters were analysed: distance travelled, mean speed (m/s), max speed (m/s), overall mobility, as well as time spent in the peripheral zones and in the centre of the test apparatus. Mice were euthanized by being placed in a CO_2_ chamber. 

### 2.3. Chemicals and Antibodies

The following chemical were used: Triton X-100 (tx, #T9284, Sigma-Aldrich, Darmstadt, Germany), bovine serum albumin (BSA; #10735086001, Sigma-Aldrich, Darmstadt, Germany), Hoechst 33342 (1:10,000, #62249, Thermo Scientific, Waltham, MA, USA). Dilutions for all antibodies were optimised as necessary and used at the concentrations indicated. A list of primary antibodies as well as their concentrations used were as follows: rabbit anti-MBP (1:1000, #AB40390, Abcam, Cambridge, UK), mouse anti-MOG (1:1000, #MAB5680, Millipore, Darmstadt, Germany), rabbit-anti-dMBP (1:1000, #AB5864, Millpore, Darmstadt, Germany), chicken anti-NFH (1:1000, #AB5539, Abcam), chicken anti-GFAP (1:1000, #AB4674, Abcam), mouse anti-vimentin (1:1000, #sc-373717, Santa Cruz, Heidelberg, Germany;), rabbit anti-Iba1 (1:1000, #019-19741, Wako, Neuss, Germany), mouse anti-SMI32 (1;1000, #ME1023, Millipore). A list of secondary antibodies as well as their concentrations used were as follows: goat anti-rabbit Alexa 488 (1:1000, #A11008, Invitrogen, Waltham, MA, USA), goat anti-chicken Alexa 633 (1:1000, #A11008, Invitrogen, Waltham, MA, USA), goat anti-mouse Dylight 549 (1:1000, #610-142-121, Thermo Scientific, Waltham, MA, USA).

### 2.4. Immunohistochemistry

Animals were euthanized with CO_2_ and perfused transcardially with ice-cold phosphate-buffered saline (PBS) and immunocytochemistry conducted as previously described [8,13]. Brain hemispheres were separated, and one half was postfixed in 4% PFA followed by cryoprotection in 30% sucrose at 4 °C. They were then snap-frozen in isopentane and stored at −80 °C, before being prepared for immunohistochemistry (IHC). The other hemisphere was further split into cortex and cerebellum and tissue was kept in RIPA buffer and protease inhibitors and stored at −80 °C. Frozen sections for IHC were cut sagitally at 12 μm thickness and were prepared exactly as described previously [8]. Briefly, cryosections were equilibrated to room temperature, rehydrated and permeabilized with 0.1% Triton X-100 (Tx; catalog#T9284, Sigma-Aldrich, Darmstadt, Germany) in PBS. No further antigen retrieval was performed. Sections were then blocked with 10% bovine serum albumin (BSA; catalog#10735086001, Sigma-Aldrich, Darmstadt, Germany) for 2 h at room temperature, before incubation with primary antibodies in PBS containing 2% BSA and 0.5% Tx at 4 °C overnight. Secondary antibody incubations were performed overnight at 4 °C in PBS supplemented with 2% BSA and 0.05% Tx. A list of primary and secondary antibodies as well as their concentrations used can be found in the section above. A counterstain with Hoechst 33342 (1:10,000, #62249, Thermo Scientific, Waltham, MA, USA) labelling the nucleus was performed at the end of the immunofluorescence protocol, where appropriate. Slices were mounted and coverslipped on microscope slides and stored in the dark before being imaged. 

### 2.5. Whole mount Staining of Sciatic Nerves

Sciatic nerves were carefully removed and fixed in 4% paraformaldehyde (PFA) over night at 4 °C. Following fixation, nerve samples were washed three times in PBS, each wash lasting 10 min, in a 24-well plate on an orbital shaker at room temperature. Nerves were then permeabilised and blocked overnight in 10% BSA and 1% TritonX-100 at 4 °C. Primary antibodies were diluted in final concentration of 10% BSA and 0.5% 1% TritonX-100 in PBS at a 1:1000 dilution for myeline basic protein (MBP) and 1:500 dilution for neurofilament H (NFH) and incubated with the nerve samples for 72 h at 4 °C in an Eppendorf tube rotator. Following primary antibody incubation, nerves were rinsed in three 15 min washes with PBS at room temperature. Nerve samples were then washed using a further 6–7 changes of PBS, with each wash lasting 1 h, in order to ensure complete removal of unbound primary antibody. Nerve samples were then incubated for 48 h at 4 °C with fluorescently labelled secondary antibodies as well as Hoechst dye in final concentration of 10% BSA and 1% TritonX-100 in PBS. Excess antibodies were removed by washing nerve samples in PBS for 6 h at room temperature, changing the buffer each hour. Following staining procedures, nerve samples were cleared in increasing glycerol concentrations at 25%, 50% and 75% (*v*/*v*) glycerol in PBS with incubations lasting 12–24 h each. The cleared nerve tissue was then mounted on a glass slide and imaged with an Sp8 confocal microscope.

### 2.6. Fluorescence Microscopy

Data acquisition and quantification was similar to our previous studies [8,13]. Confocal images were captured using a Leica Sp8 scanning confocal microscope with 20× and 40× objectives. As per previous studies, five cerebellar slices per slide, and approximately 10–12 images were taken per slide to cover most of the total area of the cerebellum. Between 6 and 8 slides were used per treatment group making a minimum of 60 images analysed per treatment group [8]. Image acquisition settings were kept the same across different treatments per experiment. Image analysis was conducted using the software ImageJ (https://imagej.nih.gov/ij/). Regions of interest were manually selected on each image, and fluorescence intensity was averaged. Mean fluorescence intensity as measured with ImageJ was used as an arbitrary unit of measure. For Iba1 quantification, we used z-stacks to compute 3D models of Iba1-positive microglia with Imaris software (Bitplane, https://imaris.oxinst.com/) rather than measuring fluorescence intensity. The surface of those 3D models was then used to identify area and volume taken up by microglia, with the hypothesis that the amoeboid and reactive state of microglia in twitcher mice will be reflected in higher volume measurements.

### 2.7. Statistical Analysis

Experimental data was analysed and graphically represented using GraphPad Prism 8.0 Software package (GraphPAD Software, Inc., San Diego, CA, USA) as detailed before [8,13]. The normality of the data was determined using the Shapiro–Wilk test. Where appropriate, behavioural, histologic and biochemical data was analysed using the parametric two-way ANOVA with the Tukey post hoc test for multiple comparisons. Our rationale for using a Two-way ANOVA was as follows: our variables were independent (independence of observations), no significant outliers were present in our data sets, and our data set was normally distributed. If data were not normally distributed, differences between groups was assessed using the non-parametric Kruskal–Wallis followed by Dunn’s multiple comparisons test. The difference in the lifespan between twitcher and wildtype mice was examined by Kaplan–Meier log-rank analysis. Raw data sets were normalised and presented as percentages of the control group average. Unless indicated, all data is expressed as mean ±SEM, two-way ANOVA and Tukey’s post hoc test (* *p* < 0.05, ** *p* < 0.01, *** *p* < 0.001, **** *p* < 0.0001). We note that for *p* values < 0.01, 0.001, this may not reflect the means are very different, and more so that the mean SD values are small. We examined every possible comparison between the study groups. Only groups that were found to be statistically different are indicated. Those not found statistically different have not been indicated.

## 3. Results

### 3.1. Fingolimod Treatment Increases Longevity of Twitcher Mice

**Figure 1:**
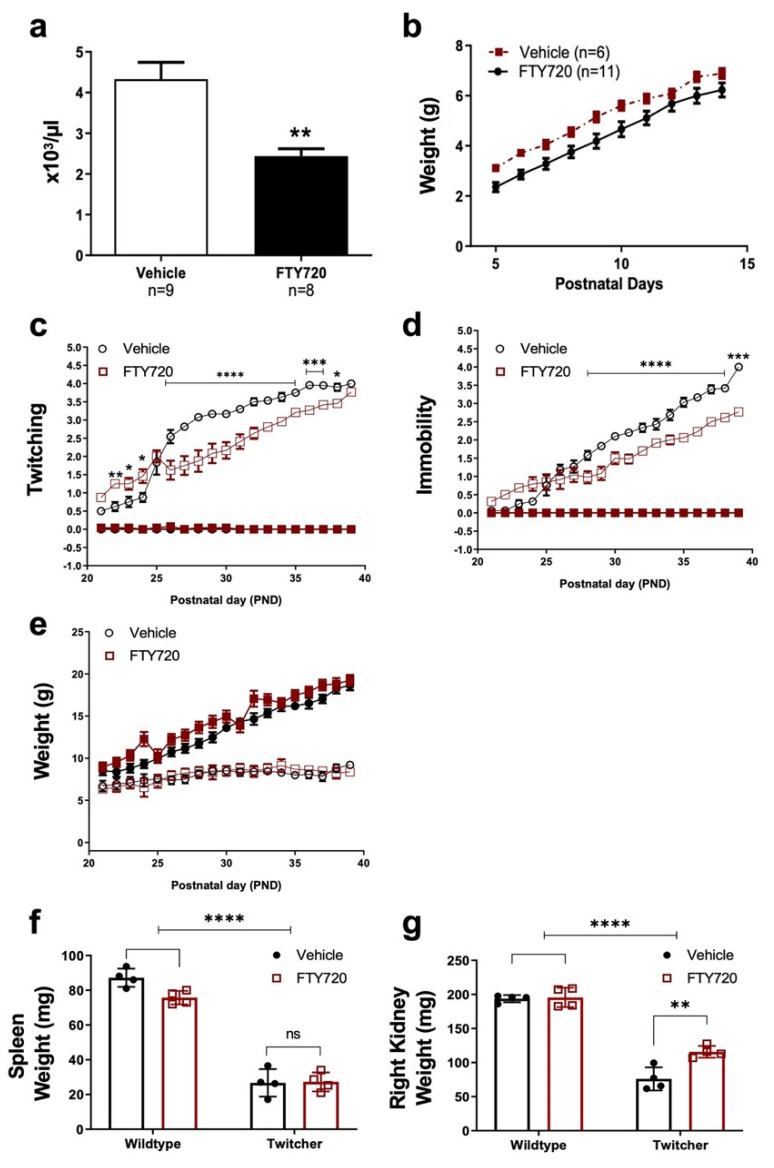
Fingolimod improves clinical phenotype in Twitcher mice. (**a**) Lymphocyte counts were performed using the Sysmex KX-21N immediately following blood collection from wildtype pups (** *p* = 0.0014, two-tailed *t*-test). (**b**) Pre-weaning body weight of fingolimod-treated wildtype pups gradually increased over the 10-day treatment period, with no significant difference compared with vehicle-treated animals. The (**c**) twitching, (**d**) mobility and (**e**) weight were statistically assessed using a two-way ANOVA followed by a Bonferroni adjustment for multiple comparisons. The number of animals per group were: vehicle (n = 15) and fingolimod (n = 15) treated wildtype mice and vehicle (n = 13) and fingolimod (n = 12) treated twitcher mice. The weight measurements of peripheral organs (**f**) spleen and (**g**) right kidney are shown. The wildtype animals in (**b**–**e**) are indicated by closed red squares. * *p* < 0.05, ** *p* < 0.01, *** *p* < 0.001, **** *p* < 0.0001.

Fingolimod was administered via suckling pipette daily between postnatal day (PND) 5 and 14. In a pilot study using wildtype animals, fingolimod treatment decreased lymphocyte numbers at PND14 (Figure 1a). No overt signs of fingolimod-related adverse effects were observed, with a consistent weight increase over the 10-day treatment period (Figure 1b). In twitcher mice, fingolimod improved twitching (Figure 1c) and mobility (Figure 1d) scores. Weight measurements of treated twitcher mice followed the same trends of vehicle treated twitcher animals (Figure 1e). A decrease in size and weight of visceral organs such as spleen (Figure 1f) and kidney (Figure 1g) of twitcher mice was also noted, with fingolimod increasing kidney weight in twitcher mice (Figure 1g). 

**Figure 2:**
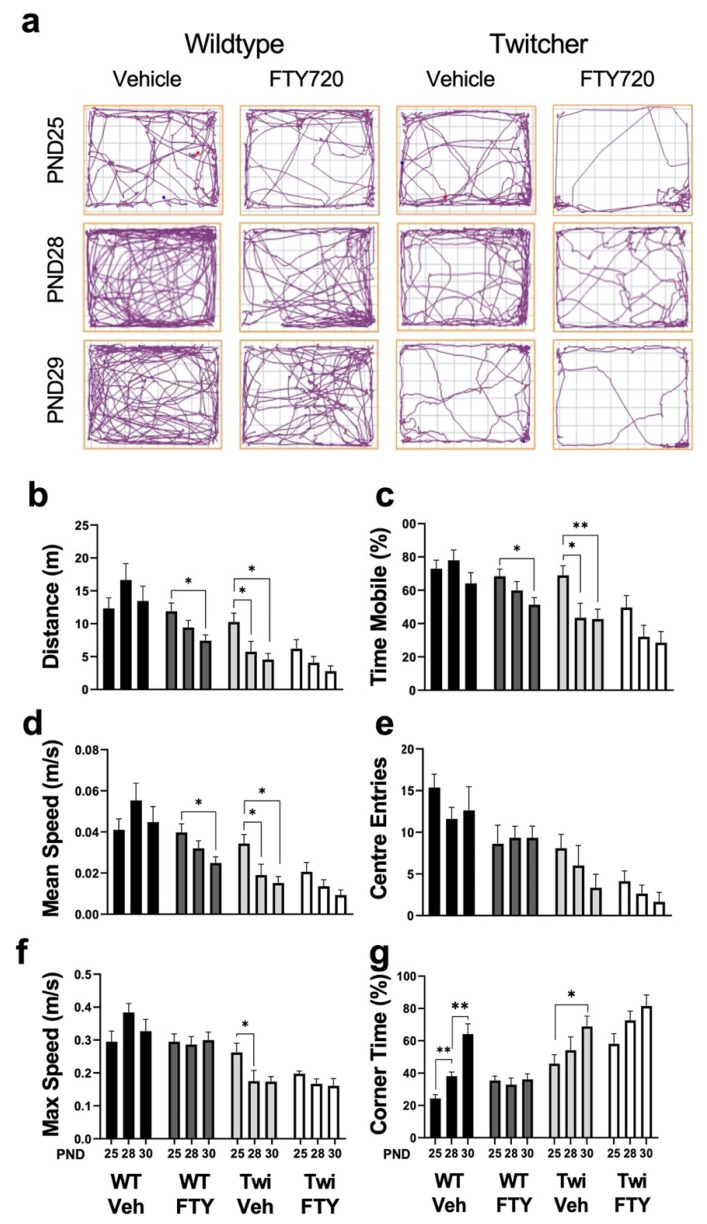
Effects of fingolimod treatment on locomotor parameters in Twitcher mice. (**a**) Representative images of open field maze tracking plots were taken at PND25, 28 and 30, following a habituation session at PND21/22. Bar graphs illustrating analysis of each locomotor parameter studied, including: (**b**) total distance travelled, (**c**) time mobile (%), (**d**) mean speed, (**e**) centre entries, (**f**) maximal speed, (**g**) time spent in corners (%). * *p* < 0.05, ** *p* < 0.01.

Tracing of animals in open field maze (Figure 2a), showed fingolimod-treated wildtype animals caused a decrease in distance travelled (Figure 2b), overall mobility (Figure 2c), and mean speed (Figure 2d). In vehicle-treated twitcher mice, we observed a decrease in almost all locomotor parameters tested over the 6 days of testing, with fingolimod exhibiting no significant improvements (Figure 2a–g). Wildtype animals spent more time in the corners as they aged from PND25 to PND30, which was attenuated by fingolimod (Figure 2g). 

**Figure 3:**
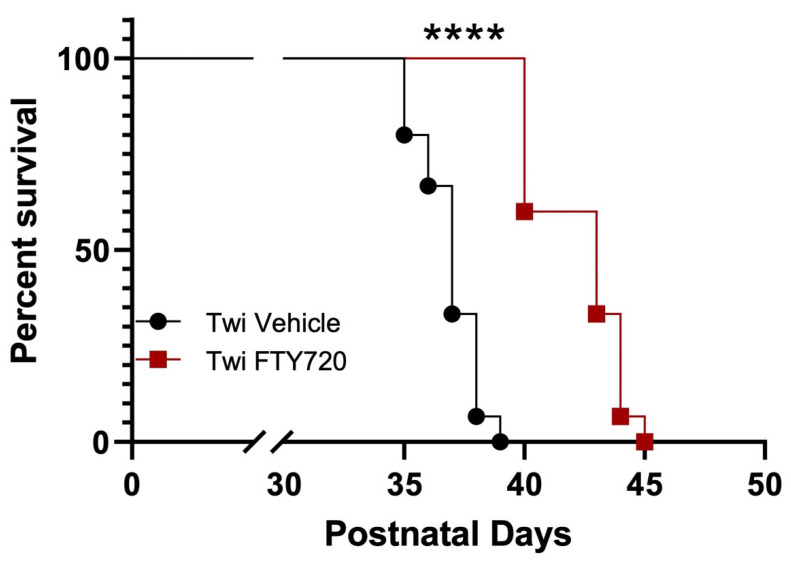
Fingolimod increases lifespan in Twitcher mice. The Kaplan–Meier survival curve demonstrates that fingolimod compared to vehicle treatment increases lifespan of twitcher mice. A log-rank test reveals a significant difference in survival distribution. **** *p* < 0.0001.

Lastly, a Kaplan–Meier survival curve showed that fingolimod increased the life span of treated compared to untreated twitcher mice (Figure 3). 

### 3.2. Early Administration of Fingolimod (FTY720) Reduces Myelin Debris in Twitcher Mice

**Figure 4:**
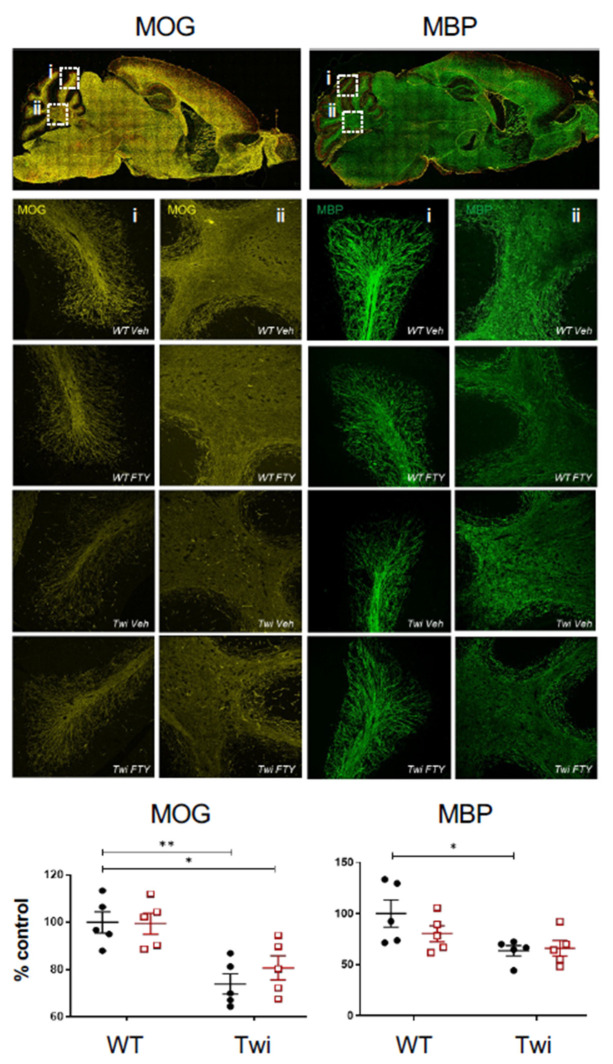
Effects of Fingolimod on levels of MBP or MOG in the cerebellum of twitcher mice when administered at neonatal stages. Representative whole parasagittal images of MOG (yellow) and MBP (green), and magnified images (dotted box) of lobes (**i**) and white matter (**ii**) areas of the cerebellum. Quantification of MOG and MBP fluorescence show in graphs (n = 5 per group). Animals were analysed between PND40 and 45. * *p* < 0.05, ** *p* < 0.01.

**Figure 5:**
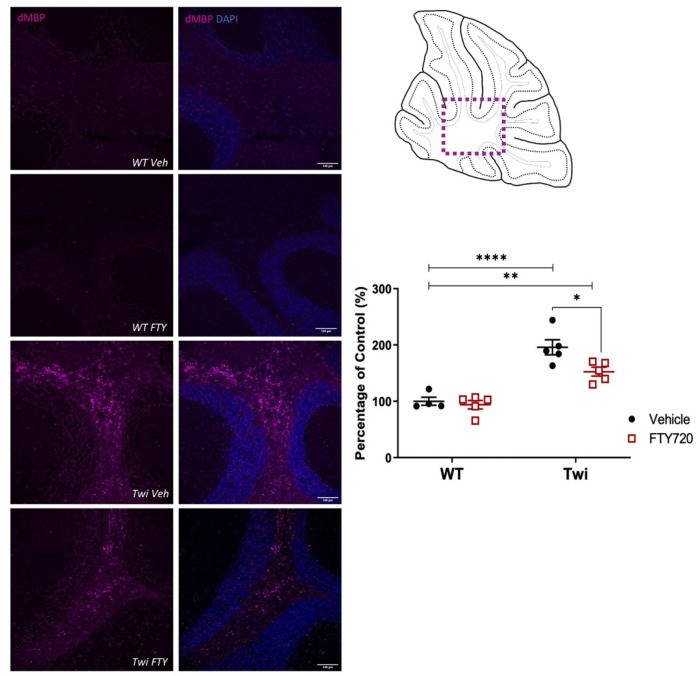
Fingolimod reduces expression of degraded MBP (dMBP) in the cerebellum of twitcher mice. Degraded MBP (dMBP) (purple) images in cerebellar white matter as depicted in (i) cartoon diagram (dotted box). Quantification of dMBP fluorescence show in graphs (n = 4–5 per group). Using an Sp8 confocal, Z-stacks were taken at 0.68 µm intervals and flattened. Scale bar, 100 µm (20× magnification). Animals were analysed between PND40 and 45. * *p* < 0.05, ** *p* < 0.01, **** *p* < 0.0001.

Our previous study treating twitcher mice with fingolimod from PND21 onwards increased the expression of myelin basic protein (MBP), with no effects on myelin oligodendrocyte protein (MOG) [8]. Here, we evaluated if earlier treatment of fingolimod from PND5 onwards could enhance the myelination state in twitcher animals. In cerebellum of twitcher mice, the fluorescence of MOG and MBP significantly decreased (Figure 4). In these cases, fingolimod administration (1 mg/kg/day) from PND5 onwards did not alter myelin levels (Figure 4). In contrast, when using an antibody against an epitope of degraded MBP (dMBP), which becomes exposed in myelin debris, extensive myelin debris clusters were seen in cerebellar white matter tracts of twitcher mice compared to wildtype animals. These myelin debris clusters were significantly reduced by fingolimod in twitcher mice (Figure 5).

### 3.3. Fingolimod (FTY720) Regulates Astrocyte and Microglia Reactivity and Axonal Damage in Twitcher Mice

**Figure 6:**
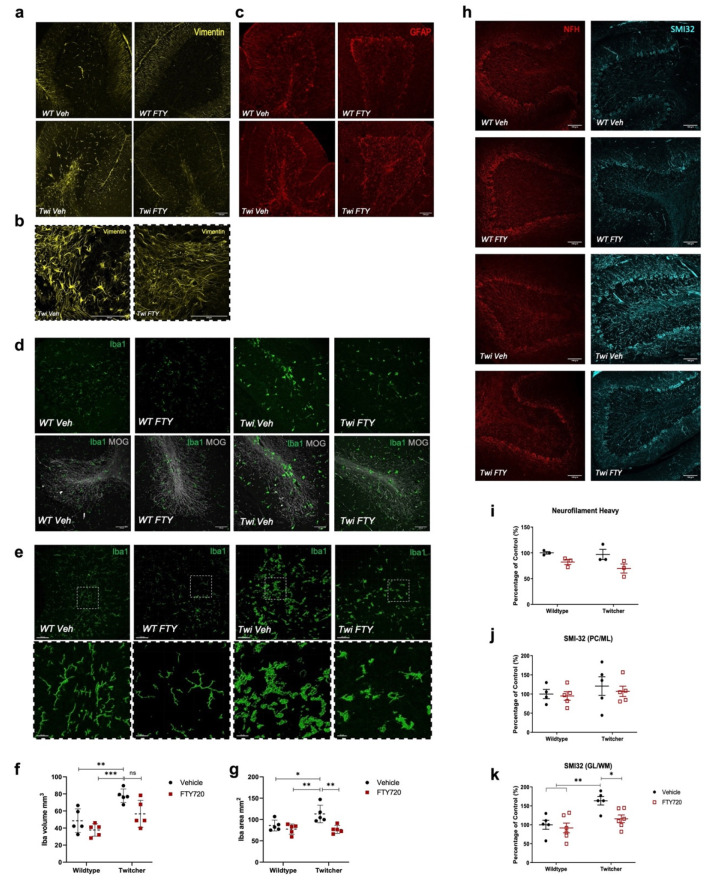
Fingolimod regulates Vimentin, Iba1 and SMI-32 fluorescence in the cerebellum of twitcher mice. (**a**) Representative images of vimentin positive fibrous astrocytes in the white matter (WM) parenchyma and Bergmann glia in the molecular layer of the cerebellum. (**b**) Images magnified at region depicted in the dotted box. (**c**) Representative images of GFAP (red) fluorescence and (**d**) Representative images of Iba1-positive microglia/macrophages (green) and MOG (grey). (**e**) Three-dimensional reconstruction of Iba1 immunofluorescent microglia using Imaris bitplane. Graphs showing (**f**) Iba1-positive volume (µm^3^) and (**g**) Iba1-positive area (mm^2^). (**h**) Representative images of NFH (red) and SMI-32 (cyan). Graphs showing (**i**) NFH fluorescence, (**j**) SMI-32 fluorescence in Purkinje cell and molecular dendritic layers (PC/ML) and (**k**) SMI-32 expression in cerebellar granular layer and white matter tracts (GL/WM). Confocal images taken at 20× or 40× magnification. Scale bar, 100 µm (**a**,**b**,**c**,**h**). Scale bar inserts, 20 µm (**d**,**e**). Animals were analysed between PND40 and 45. * *p* < 0.05, ** *p* < 0.01, *** *p* < 0.001.

In twitcher mice, vimentin fluorescence was increased in the white matter layer of the cerebellum of twitcher mice, as seen by a disruption in the organised astrocyte scaffold alignment (Figure 6a,b). The vimentin positive astrocytes relocated and clustered around white matter tracts, which was qualitatively reduced by fingolimod (Figure 6a,b). In contrast, GFAP fluorescence showed no differences in the conditions examined (Figure 6c). Computed 3D models of z-stacks showed a significant increase in Iba1 volume (mm^3^) and covered area (mm^2^) in twitcher mice (Figure 6d), reflected by ramified and amoeboid microglia morphologies (Figure 6e). Fingolimod reduced the increase in Iba1 volume (mm^3^) and significantly attenuated the Iba1-positive area (mm^2^) in twitcher mice (Figure 6f,g). Abnormal accumulations of neurofilaments and aberrant phosphorylation were examined using the non-phosphorylated NFH epitope SMI-32 as a marker for neuronal damage (Figure 6h–k). There was a significant increase in SMI-32 immunoreactivity in granular layer (GL) and white matter tracts (WM) of twitcher mice that was significantly reduced by fingolimod (Figure 6k). 

### 3.4. Effect of Fingolimod in the Sciatic Nerve of Twitcher Mice

**Figure 7:**
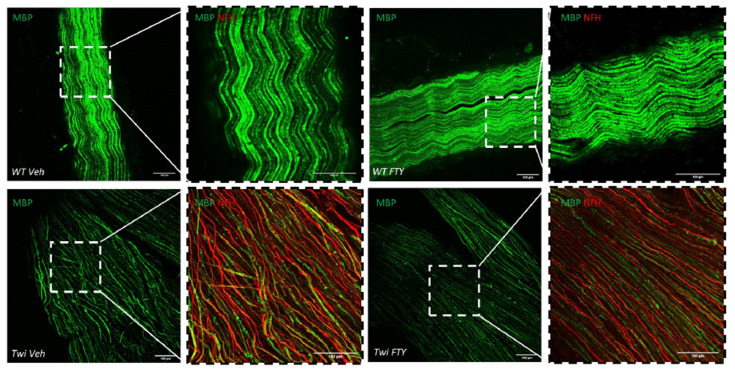
Fingolimod does not rescue demyelination in sciatic nerves. Representative images (z-stacks) of sciatic nerves with MBP (green) and NFH (red) immunofluorescence, captured using an Sp8 confocal microscope. Images magnified at region depicted in the dotted box are also shown. Scale bar, 100 µm. Animals were analysed between PND40 and 45.

In the present study, sciatic nerves of twitcher mice displayed demyelinated axons, showing little MBP expression and primarily the remaining NFH staining (Figure 7). In twitcher mice, the NFH staining was very likely due to the nerves being severely demyelinated (Figure 7). Treatment with fingolimod had no significant effects on the expression of MBP in either wildtype or twitcher mice (Figure 7). 

## 4. Discussion

Within the last decade, ample research has shown that the effects of fingolimod reach beyond that of immunomodulation and directly act within the central nervous system (CNS) [1,2,4,5,6,7,8]. Fingolimod, and selective S1PR compounds, can induce process extension, enhance cell survival and stimulate cell maturation [14,15,16]. When moving to in vivo studies, the fingolimod-mediated beneficial effects on myelin repair appear to be specific to particular animal models. For example, fingolimod failed to induce remyelination following acute and chronic cuprizone exposure [17,18,19]. It also did not significantly contribute to myelin repair following focal demyelination induced by lysolecithin injection in rat spinal cord [17]. In the present study, we administered fingolimod orally at a concentration of 1 mg/kg/day, starting at postnatal day 5 (PND5). As expected, myelin levels decreased in twitcher mice with extensive myelin debris observed in white matter. While fingolimod did not alter MBP and MOG expression, treatment with this drug significantly reduced myelin debris in twitcher mice. A differential developmental expression of S1PRs and the opposing roles they play in oligodendrocyte cell lineages during development, as previously reported [15,20,21], may in part explain the effects of fingolimod on myelination and myelin debris clearance. 

The neuroprotective spectrum of fingolimod has been illustrated in various in vivo and in vitro studies [19,22]. The relationship between axonal damage and remyelination is complex, and may occur independently [19,23]. Studies have supported S1P1R-mediated neuroprotection by fingolimod in vitro in cortical primary and organotypic slice cultures exposed to NMDA excitotoxicity, as well as in animal models of Parkinson’s disease and kainic acid neurodegeneration [22,24]. In agreement, we show fingolimod to improve axonal integrity (or protection) independent of myelination levels, where axonal damage, quantified by SMI32, was reduced by fingolimod in twitcher mice. In addition to neuroprotection via direct S1PR modulation on neurons, the observed dampened axonal damage in our study could also be linked to the concurrent decrease in myelin debris. It is well established that myelin debris accumulation inhibits axonal regeneration and remyelination [25,26].

Here, we show that twitcher mice exhibit a substantive increase in microglial volume and a morphological change from a ramified to an amoeboid state accumulating in white matter tracts, which mirrors results from numerous previous experiments [27,28,29,30]. Fingolimod administration partially reversed the reactive morphology of microglia in our study, which was reflected by a decrease in volume of Iba1-positive cells. This finding agrees with ex vivo results from organotypic slice cultures, reporting a decrease in phagocytosing microglia with fingolimod accompanied by an increase in microglia numbers [31]. Further in vivo findings have shown similar effects where fingolimod reduces neurodegeneration as well as microglial activation in a kainic acid-induced model of neuronal death and inflammation [22]. 

In twitcher mice, astrocytes undergo morphological changes associated with activation, with evident astrogliosis in the hindbrain and cerebellum of twitcher mice as early as 3 weeks of age [30]. While GFAP immunofluorescence did not change between groups, vimentin positive astrocytes relocated and clustered around white matter tracts, areas of demyelination and axonal damage in twitcher mice, and these relocated astrocytes were reduced by fingolimod. These results are in accordance with previous studies describing extensive astrogliosis surrounding white matter tracts in twitcher mice [30,32], and in line with fingolimod regulating astrocyte migration [2]. In vitro, fingolimod regulates a number of signalling pathways in both rodent and human astrocytes, where S1P receptor drugs attenuate psychosine-mediated apoptosis of astrocytes [6].

While psychosine exerts its cytotoxic effects within the brain [33,34,35], the pathology of KD also extends to peripheral organs with increased psychosine accumulation [9,12]. In sciatic nerves of twitcher mice, peripheral neuropathy led to abnormalities in myelin as early as 1 week postnatal [10,11]. In agreement, we found sciatic nerves showed severe reductions of myelin, however, our results did not reveal any beneficial effects of fingolimod. While fingolimod has previously been shown to promote peripheral axon regeneration and demyelination following sciatic nerve crush injury, our findings sit in line with no fingolimod-mediated effect on myelination in neuronal and Schwann cell co-cultures [36]. Overall, these findings highlight a differential effect of fingolimod in the central and peripheral nervous systems, at least in twitcher animals. Behaviourally, the early administration of fingolimod in twitcher mice modestly improved twitching and immobility scores as well as lifespan, albeit without reducing weight loss and altered locomotor activity. The lack of observed effects of fingolimod in the peripheral nervous system and visceral organs may in part explain the modest effects of this drug on lifespan observed in this study.

## Data Availability

The data that support the findings of this study are available from the corresponding author upon reasonable request.
